# Supplementary figures and images for: Micronutrient-deficient diets and possible environmental enteric dysfunction in Buruli ulcer endemic communities in Ghana: Lower dietary diversity and reduced serum zinc and vitamin C implicate micronutrient status a possible susceptibility factor
Source: PLoS Negl Trop Dis. 2025 Mar 12;19(3):e0012871. doi: 10.1371/journal.pntd.0012871 (PMC11902277; doi:10.1371/journal.pntd.0012871)

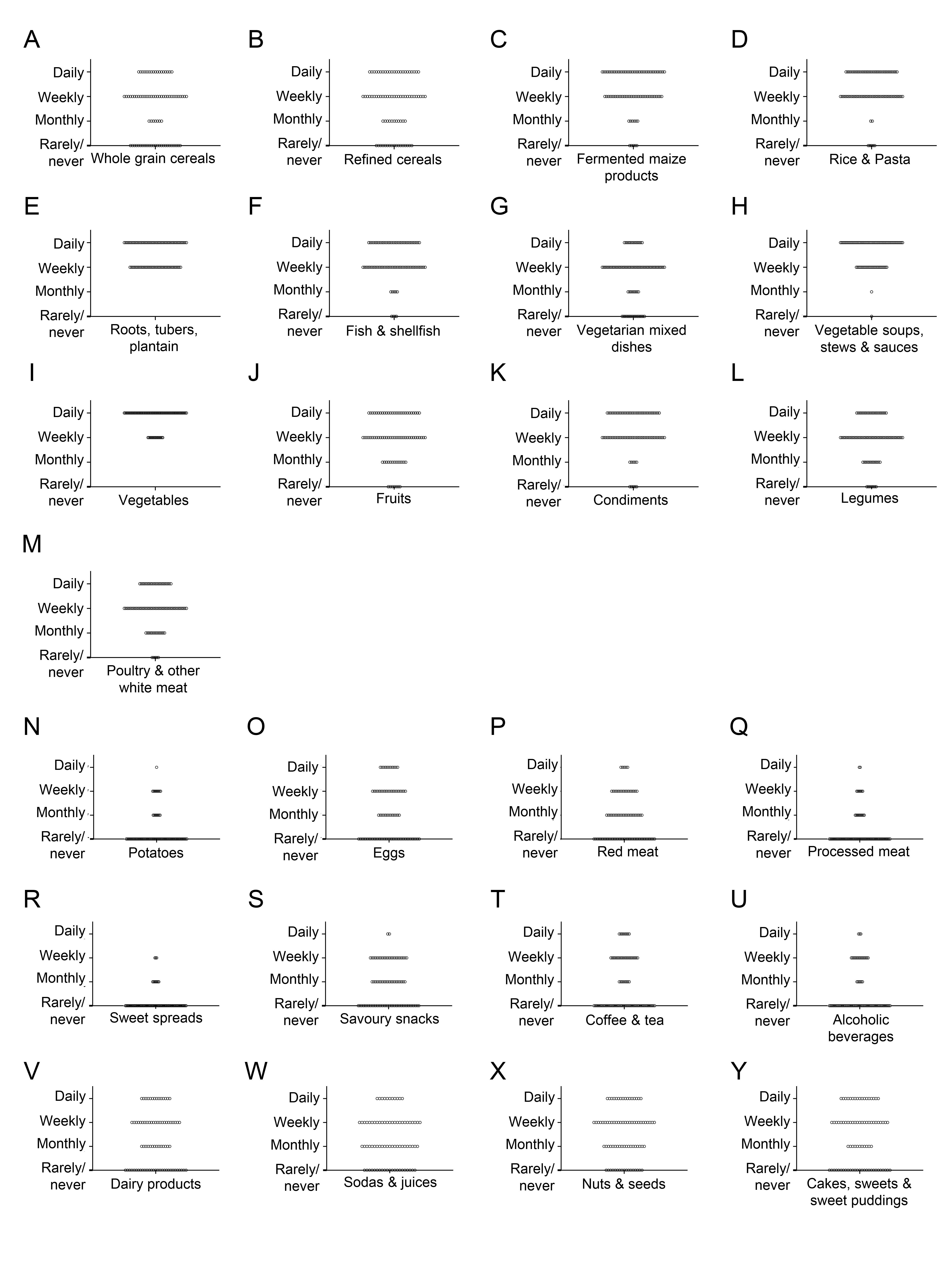

Supplement: S1 Fig — A food frequency questionnaire was put to all 80 Cohort 1 participants (40 BU cases, 40 controls), covering a total of 105 foods from 25 food groups. There are 4 possible answers for frequency of consumption for each food, namely daily, weekly, monthly, and rarely/never. This figure visualises the distribution of the most frequent consumption of one or more foods in that food group in the past month amongst the participants. The red line represents the frequency of consumption, and its length is proportional to the number of participants with that maximal frequency. Data from the food groups showed that at least 60% of the participants ate foods from these food groups at least weekly, where similar numbers consumed foods monthly, rarely or never vs. weekly or daily (N-Q), and those where at least 60% of the participants ate them monthly, rarely or never (R-Y). (TIF) [file pntd.0012871.s001.tif]

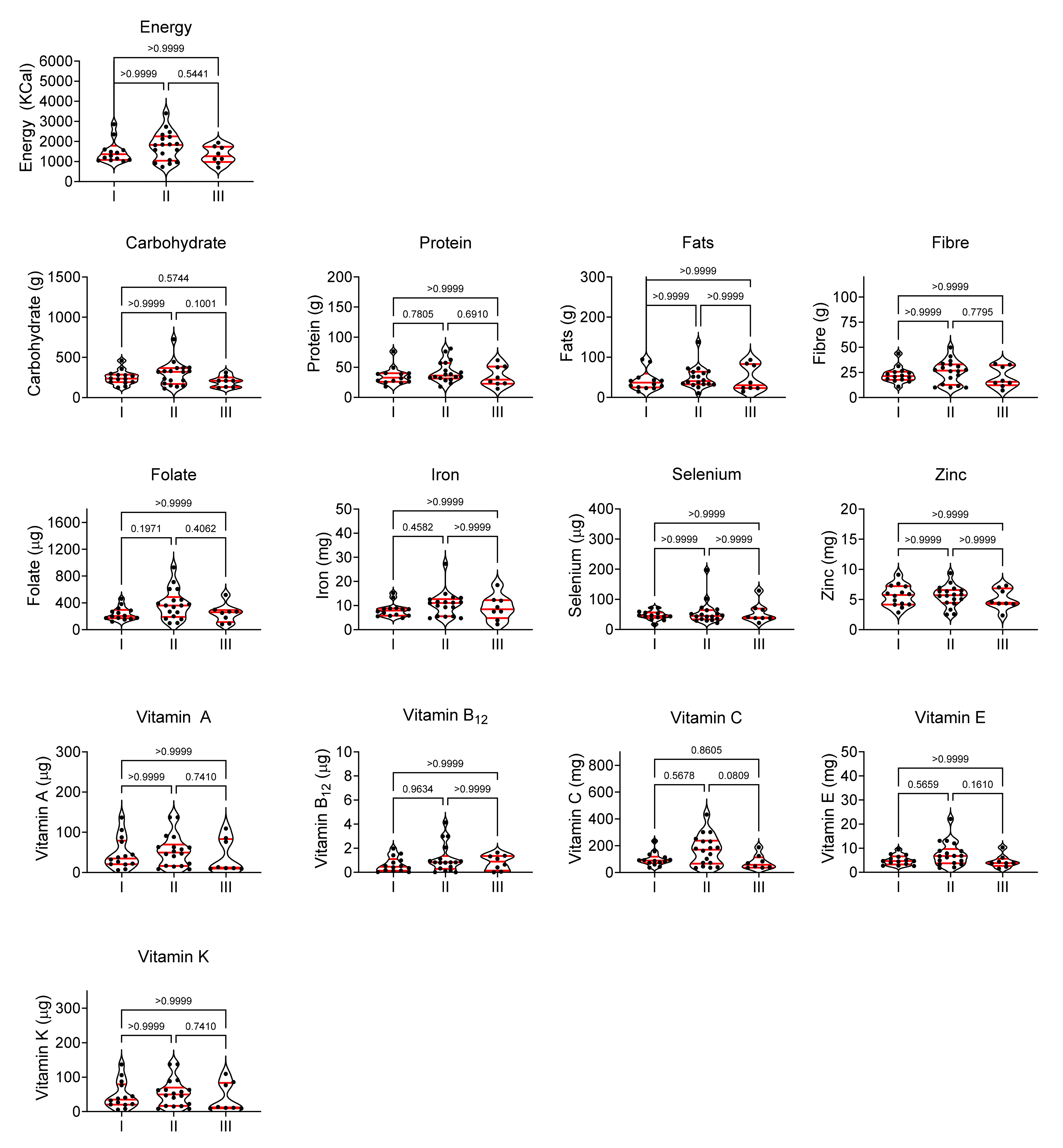

Supplement: S2 Fig — Serum concentrations of vitamin C, zinc and IL-6 were analysed using Spearman’s correlation, and are presented as a heatmap. Spearman’s correlation coefficients are given along with P-value (in brackets). (TIF) [file pntd.0012871.s002.tif]

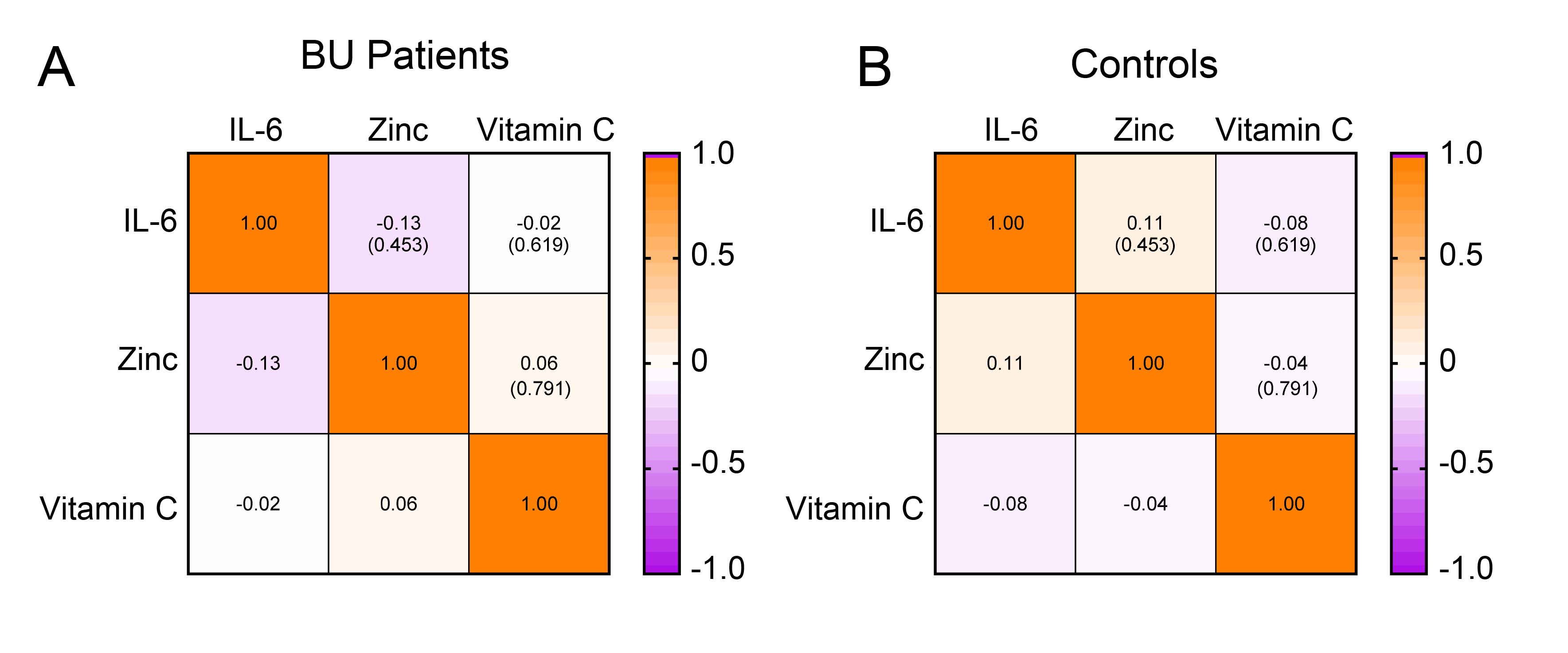

Supplement: S3 Fig — Serum concentrations of vitamin C, zinc and IL-6 were analysed using Spearman’s correlation, and are presented as a heatmap. Spearman’s correlation coefficients are given along with P-value (in brackets). (TIF) [file pntd.0012871.s003.tif]
