# Supplementary material for: Micronutrient-deficient diets and possible environmental enteric dysfunction in Buruli ulcer endemic communities in Ghana: Lower dietary diversity and reduced serum zinc and vitamin C implicate micronutrient status a possible susceptibility factor
Source: PLoS Negl Trop Dis. 2025 Mar 12;19(3):e0012871. doi: 10.1371/journal.pntd.0012871 (PMC11902277; doi:10.1371/journal.pntd.0012871)
Supplement: S1 Table — (DOCX) [file pntd.0012871.s004.docx]

**S1 Table. List of foods used in the FFQ administration.**

| **Food group** | **Food items** |
| --- | --- |
|  |  |
| Whole grain cereals | Oats porridge, maize porridge, millet porridge, wheat porridge, whole meal bread/roll, Tuo zafi (T.Z.) |
| Refined cereals | Sugar coated cereals, cornflakes, white bread/roll, pancake, flour chips/achumo, kaafa |
| Fermented maize products | Kenkey, Banku, Akple |
| Rice and pasta | White rice cooked, local rice cooked, noodles, macaroni |
| Roots, tubers and plantain | Yam, cassava or cocoyam, plantain, fufu, kokonte, gari |
| Potatoes | Boiled instant potatoes/oven baked potatoes, fried potatoes chips, roast potatoes |
| Nuts and seeds | Cashew nuts, groundnut/pea nuts, almond/hazelnut, pistachio, coconut, sultana/raisins/currants |
| Legumes | Kidney beans, soya beans, black eyed beans, baked beans, chicks peas, runner/green beans, ground nut soup |
| Dairy products | Milk, full fat yoghurt, full fat cheese, low fat cheese, butter/margarine, cocoa, horlicks, milkshake/fula |
| Eggs | Egg boiled/poached, egg fried/omelette |
| Red meat | Beef/lamb/pork, bush meat, rabbit |
| Poultry/other white meat | Chicken/other poultry, snail |
| Processed meat | Burger, bacon, ham, sausage/luncheon meat/corned beef |
| Fish *and shellfish* | Fish, fish fingers, fish fried in butter, crabs, prawn, octopus |
| Vegetarian mixed dishes | Jollof rice, fried rice, waakye, soya bean curd/tofu, beans stew |
| Vegetable soups, stews, sauces | Tomato stew, melon seed and spinach stew, palmnut soup, okro soup/stew, light soup, kontomire (cocoyam leaves) |
| Vegetables | Sweet corn, okro/nkruma, garden egg/aubergine, avocado, garden peas/peas, mushroom, broccoli/cauliflower, carrot, cabbage, brussels sprouts, ayoyo, spring onions, onions, tomatoes, tin-tomato, sweet peppers, lettuce, cucumber, coleslaw, beetroot, green pepper, Ghanaian green pepper (Kpakposhitor), |
| Fruits | Banana, pears, pomegranates, mangoes, pineapple, grapefruits, orange/satsuma, grapes, melon, apple, peach/plum/nectarine, strawberries/cherries, pawpaw |
| Sweet spreads | Jam, marmalade, honey, peanut butter |
| Cakes and sweets, and sweet puddings | Sugar added to cereals, tea, coffee, cakes/scones/doughnuts, ice cream/frozen desserts, sweets biscuits, chocolate coated sweet biscuits, chocolate/chocolate bar, custard, rice pudding, sweets, toffees, mints |
| Savoury snacks | Crisp/pocket snacks, fried plantain chips, salty biscuits, regular popcorn, buttered popcorn |
| Condiments | Hot pepper sauce, tomato ketchup, salad pepper (powdered) |
| Alcoholic beverages | Beer/larger/cider, port/sherry/liqueur, spirits, pito/solom, palmwine (alcohol) |
| Sodas and juices | Real fruit juice, fruit squash, fizzy soft drink, low calorie fizzy drink, bissap (sobolo), liha (sprout maize), palmwine (fresh) |
| Coffee and tea | Tea, Coffee |
|  |  |
