# Supplementary material for: Micronutrient-deficient diets and possible environmental enteric dysfunction in Buruli ulcer endemic communities in Ghana: Lower dietary diversity and reduced serum zinc and vitamin C implicate micronutrient status a possible susceptibility factor
Source: PLoS Negl Trop Dis. 2025 Mar 12;19(3):e0012871. doi: 10.1371/journal.pntd.0012871 (PMC11902277; doi:10.1371/journal.pntd.0012871)
Supplement: S2 Table — (DOCX) [file pntd.0012871.s005.docx]

**S2 Table. Food groups used in the Dietary Diversity Score, generated from 24-hour recall data.**

| cereals | roots & tubers | green leafy vegetables |
| --- | --- | --- |
| other vegetables | fruits | meat |
| fish & seafood | eggs | pulses & nuts |
| milk & milk products | oils & fats | sugar |
| condiments | drinks & beverages | ‘other’ |
