# Supplementary material for: Micronutrient-deficient diets and possible environmental enteric dysfunction in Buruli ulcer endemic communities in Ghana: Lower dietary diversity and reduced serum zinc and vitamin C implicate micronutrient status a possible susceptibility factor
Source: PLoS Negl Trop Dis. 2025 Mar 12;19(3):e0012871. doi: 10.1371/journal.pntd.0012871 (PMC11902277; doi:10.1371/journal.pntd.0012871)
Supplement: S3 Table — a; T χ² test. p-value < 0.05 was statistically significant. (DOCX) [file pntd.0012871.s006.docx]

**S3 Table. Socioeconomic status of Cohort 1 study participants.**

|  | **Cohort 1** | | |
| --- | --- | --- | --- |
| **Variables** | **BU cases** | **Controls** | **P-value** |
|  | n=40 | n=40 |  |
| **Education** |  |  | 0.236^a^ |
| *Primary* | 24 (58.5%) | 18 (45%) |  |
| *Secondary* | 8 (19.5%) | 16 (40%) |  |
| *Tertiary* | 0 | 2 (5%) |  |
| *None* | 9 (22%) | 4 (10%) |  |
| **Marital Status** |  |  | 0.769^a^ |
| Schooling | 20 (50%) | 22 (55%) |  |
| *Single* | 6 (15%) | 6 (15%) |  |
| *Married* | 13 (33%) | 12 (30%) |  |
| *Divorced* | 1 (3%) | 0 |  |
| **Household monthly income** |  |  | 0.478^a^ |
| *<GH₵ 50* | 6 (15%) | 4 (10%) |  |
| *GH₵ 50-200* | 10 (25%) | 15 (38%) |  |
| *GH₵201-800* | 23 (58%) | 21(53%) |  |
| *>GH₵800* | 0 | 0 |  |
| **Occupation** |  |  |  |
| *Formal* | 0 | 0 | 0.236^a^ |
| *Informal* | 15 (38%) | 17 (43%) |  |
| *Attending School* | 20 (50%) | 22 (55%) |  |
| *Unemployed* | 5 (13%) | 1 (3%) |  |

^a^; T χ² test. p-value < 0.05 was statistically significant.
