# Supplementary material for: Micronutrient-deficient diets and possible environmental enteric dysfunction in Buruli ulcer endemic communities in Ghana: Lower dietary diversity and reduced serum zinc and vitamin C implicate micronutrient status a possible susceptibility factor
Source: PLoS Negl Trop Dis. 2025 Mar 12;19(3):e0012871. doi: 10.1371/journal.pntd.0012871 (PMC11902277; doi:10.1371/journal.pntd.0012871)
Supplement: S4 Table — Unit conversions: 1g of carbohydrate is equivalent to 4 Kcal, 1g of protein is equivalent to 4 Kcal and 1g of fats is equivalent to 9 Kcal, which were then used to calculate percentages. (DOCX) [file pntd.0012871.s007.docx]

**S4 Table. Mean percentage of macronutrients in the diet of Cohort 1 participants.**

|  |  |  |  |
| --- | --- | --- | --- |
| **Nutrients** | **Mean nutrient intake (%)** | | |
|  | **All** | **BU Cases** | **Controls** |
|  | N=80 | N=40 | N=40 |
|  |  |  |  |
| Energy (Kcal) | 1629 (100%) | 1594 (100%) | 1664 (100%) |
| Carbohydrate (g) | 292 (64.4%) | 261.9 (64.4%) | 322.7 (67.9%) |
| Protein (g) | 42.2 (9.6 %) | 38.84 (9.6%) | 45.63 (9.6%) |
| Fats (g) | 23.5 (24.0%) | 46.99 (26.0%) | 47.52 (22.5%) |
|  |  |  |  |

Unit conversions: 1g of carbohydrate is equivalent to 4 Kcal, 1g of protein is equivalent to 4 Kcal and 1g of fats is equivalent to 9 Kcal, which were then used to calculate percentages.
