# Supplementary material for: Micronutrient-deficient diets and possible environmental enteric dysfunction in Buruli ulcer endemic communities in Ghana: Lower dietary diversity and reduced serum zinc and vitamin C implicate micronutrient status a possible susceptibility factor
Source: PLoS Negl Trop Dis. 2025 Mar 12;19(3):e0012871. doi: 10.1371/journal.pntd.0012871 (PMC11902277; doi:10.1371/journal.pntd.0012871)
Supplement: S5 Table — Data were compared in cases and controls between males and females using a Mann-Whitney test. P-value <0.05 are indicated in bold text. (DOCX) [file pntd.0012871.s008.docx]

**S5 Table. Comparison of energy and nutrient intake of Cohort 1 study participants between sexes.**

|  |  |  |  |  |  |
| --- | --- | --- | --- | --- | --- |
| **Nutrients** | **Sex** | **BU Cases** | **P-value** | **Controls** | **P-value** |
|  |  | Male N=19; Female N=21 |  | Male N=22; Female N=18 |  |
|  |  | Median (range) |  | Median (range) |  |
|  |  |  |  |  |  |
|  |  |  |  |  |  |
| Energy (Kcal) | Male | 1683.0 (704.4-3403) | 0.551 | 1779.0 (693.9-3315) | 0.172 |
|  | Female | 1481.0 (733.3-2860) |  | 1540.0 (812-2527) |  |
|  |  |  |  |  |  |
| Carbohydrate (g) | Male | 281.6 (119.2-726.1) | 0.291 | 296.7 (91.9-2331) | 0.058 |
|  | Female | 232.0 (112.2-459.9) |  | 244.3 (108.6-406.7) |  |
|  |  |  |  |  |  |
| Protein (g) | Male | 33.64 (14.5-76.2) | 0.533 | 45.8 (8.1-89.3) | 0.381 |
|  | Female | 34.2 (18.0-81.1) |  | 38.9 (11.6-111.3) |  |
|  |  |  |  |  |  |
| Fats (g) | Male | 33.7 (14.6-88.8) | 0.184 | 44.9 (20.3-104.2) | 0.459 |
|  | Female | 42.9 (10.6-137.9) |  | 43.9 (18.7-79.3) |  |
|  |  |  |  |  |  |
| Fibre (g) | Male | 20.8 (7.2-49.8) | 0.763 | 26.0 (7.6-49.9) | 0.262 |
|  | Female | 25.5 (9.9-43.8) |  | 19.5 (9.4-45.5) |  |
|  |  |  |  |  |  |
| Folate (µg) | Male | 267.4 (79.5-928.8) | 0.995 | 343.7 (4.2-916.5) | **0.018** |
|  | Female | 298.4 (96.8-710.1) |  | 214.6 (61.8-475.1) |  |
|  |  |  |  |  |  |
| Iron (mg) | Male | 9.93 (2.2-18.5) | 0.733 | 11.2 (3.7-23.7) | 0.100 |
|  | Female | 8.9 (4.7-27.3) |  | 9.3 (4.2-18.6) |  |
|  |  |  |  |  |  |
| Selenium (µg) | Male | 46.5 (16.9-128.8) | 0.683 | 77.0 (28.4-176.8) | 0.240 |
|  | Female | 43.3 (29.0-197.3) |  | 66.7 (13.3-231.1) |  |
|  |  |  |  |  |  |
| Vitamin A (µg) | Male | 423.1 (6.0-1852) | 0.587 | 437.5 (12.04-3778.0) | 0.155 |
|  | Female | 282.1 (7.81-2153) |  | 281.6 (17.71-1575) |  |
|  |  |  |  |  |  |
| Vitamin B_12_ (µg) | Male | 0.6 (0.0-4.1) | 0.899 | 2.0 (0.0-11.5) | 0.296 |
|  | Female | 0.8 (0.0-3.0) |  | 1.0 (0.14-8.7) |  |
|  |  |  |  |  |  |
| Vitamin C (mg) | Male | 91.9 (35.4-432.4) | 0.743 | 104.4 (23.4-247.6) | 0.209 |
|  | Female | 75.3 (33.00-301) |  | 64.3 (32.2-223.7) |  |
|  |  |  |  |  |  |
| Vitamin E (mg) | Male | 5.0 (2.3-8.9) | 0.952 | 6.2 (1.7-13.5) | 0.237 |
|  | Female | 4.8 (1.5-22.14) |  | 5.650 (2.05-8.9) |  |
|  |  |  |  |  |  |
| Vitamin K (µg) | Male | 44.5 (9.1-109.5) | 0.743 | 38.2 (4.3-211.3) | 0.396 |
|  | Female | 22.0 (5.8-137.2) |  | 28.5 (2.3-119.1) |  |
|  |  |  |  |  |  |
| Zinc (mg) | Male | 5.6 (2.4-7.8) | 0.606 | 7.4 (3.5-12.5) | 0.155 |
|  | Female | 5.9 (2.5-9.4) |  | 5.3 (1.7-14.14) |  |
|  |  |  |  |  |  |
|  |  |  |  |  |  |

Data were compared in cases and controls between males and females using a Mann-Whitney test. P-value <0.05 are indicated in bold text.
