# Supplementary material for: Micronutrient-deficient diets and possible environmental enteric dysfunction in Buruli ulcer endemic communities in Ghana: Lower dietary diversity and reduced serum zinc and vitamin C implicate micronutrient status a possible susceptibility factor
Source: PLoS Negl Trop Dis. 2025 Mar 12;19(3):e0012871. doi: 10.1371/journal.pntd.0012871 (PMC11902277; doi:10.1371/journal.pntd.0012871)
Supplement: S6 Table — Data were compared in cases and controls between those with different levels of household monthly income, using the Kruskal-Wallis test and Dunn’s post-hoc test for multiple comparisons. (DOCX) [file pntd.0012871.s009.docx]

**S6 Table: Comparison of energy and nutrient intake of Cohort 1 study participants between those with different household income.**

|  |  |  |  |  |  |
| --- | --- | --- | --- | --- | --- |
|  |  | **Household monthly income** | | | |
| **Nutrients** | **Group** | **<Gh¢50** | **Gh¢50-200** | **Gh¢201-800** | **P-value** |
|  |  | BU cases N=6 Controls N=4 | BU cases N=10  Controls N=15 | BU cases N=24 Controls N=21 |  |
|  |  | Median (range) | Median (range) | Median (range) |  |
|  |  |  |  |  |  |
| Energy (Kcal) | BU cases | 1165 (914-1876) | 1629 (926-2734) | 1525 (704-3403) | 0.690 |
|  | Controls | 1401 (694-3315) | 1732 (812-3296) | 1553 (723-2828) | 0.763 |
|  |  |  |  |  |  |
| Carbohydrate (g) | BU cases | 209.0 (136-329) | 276.0 (119-444) | 249.0 (112-726) | 0.714 |
|  | Controls | 209.0 (91.9-558) | 290.0 (109-460) | 253.0 (113-2331) | 0.712 |
|  |  |  |  |  |  |
| Protein (g) | BU cases | 28.1 (26.2-38.4) | 39.4 (23.2-61.8) | 34.5 (14.5-81.1) | 0.405 |
|  | Controls | 32.6 (8.15-73.4) | 41.2 (24.7-111) | 45.1 (11.6-75.8) | 0.684 |
|  |  |  |  |  |  |
| Fats (g) | BU cases | 45.3 (14.6-51.7) | 37.6 (22.9-138) | 36.0 (10.6-94.6) | 0.900 |
|  | Controls | 51.8 (33.8-98.1) | 42.8 (29.1-104) | 44.9 (18.7-84.0) | 0.492 |
|  |  |  |  |  |  |
| Fibre (g) | BU cases | 21.3 (10.0-33.6) | 24.2 (9.95-41.1) | 24.4 (7.17-49.8) | 0.983 |
|  | Controls | 18.3 (9.1-49.4) | 24.8 (10.6-49.9) | 21.7 (7.59-49.4) | 0.875 |
|  |  |  |  |  |  |
| Folate (µg) | BU cases | 263 (173-382) | 231 (90.5-518) | 269 (79.5-929) | 0.756 |
|  | Controls | 319 (253-512) | 327 (4.2-604) | 238 (61.8-916) | 0.200 |
|  |  |  |  |  |  |
| Iron (mg) | BU cases | 7.2 (5.5-11.4) | 10.2 (3.9-18.5) | 9.1 (2.2-27.3) | 0.633 |
|  | Controls | 6.8 (4.5-16.6) | 12.0 (4.5-19.0) | 8.9 (3.7-23.7) | 0.197 |
|  |  |  |  |  |  |
| Selenium (µg) | BU cases | 42.6 (29.0-43.0) | 50.2 (30.7-129) | 44.9 (16.9-197) | 0.126 |
|  | Controls | 76.1 (36.8-177) | 79.2 (29.8-231) | 65.7 (13.3-142) | 0.284 |
|  |  |  |  |  |  |
| Vitamin A (µg) | BU cases | 554.0 (282-1307) | 614.0 (7.82-2153) | 145.0 (6.00-2153) | 0.168 |
|  | Controls | 427.0 (31.9-1631) | 392.0 (18.1-2174) | 278 (12.0-3778) | 0.785 |
|  |  |  |  |  |  |
| Vitamin B_12_ (µg) | BU cases | 0.815 (0.100-2.99) | 0.815 (0.0-2.06) | 0.520 (0.00-4.14) | 0.975 |
|  | Controls | 1.98 (0.32-2.66) | 1.86 (0.54-9.01) | 0.94 (0.00-11.5) | 0.652 |
|  |  |  |  |  |  |
| Vitamin C (mg) | BU cases | 130 (60.0-186) | 87.0 (39.1-301) | 97.9 (33.0-432) | 0.854 |
|  | Controls | 115 (66.1-157) | 62.0 (23.4-248) | 105 (33.9-224) | 0.068 |
|  |  |  |  |  |  |
| Vitamin E (mg) | BU cases | 6.62 (2.76-7.27) | 4.87 (2.05-22.1) | 4.58 (1.50-13.1) | 0.662 |
|  | Controls | 8.1 (6.03-13.6) | 6.0 (1.69-12.0) | 5.4 (2.05-13.1) | 0.221 |
|  |  |  |  |  |  |
| Vitamin K (µg) | BU cases | 63.1 (8.29-106) | 34.9 (7.97-137) | 33.5 (5.83-137) | 0.440 |
|  | Controls | 92.7 (40.7-153) | 30.0 (4.27-195) | 27.5 (2.72-211) | 0.089 |
|  |  |  |  |  |  |
| Zinc (mg) | BU cases | 4.95 (2.55-6.16) | 5.70 (3.31-7.30) | 5.86 (2.36-9.38) | 0.298 |
|  | Controls | 5.16 (3.66-12.0) | 7.21 (3.74-14.1) | 6.24 (1.70-11.4) | 0.445 |
|  |  |  |  |  |  |
|  |  |  |  |  |  |

Data were compared in cases and controls between those with different levels of household monthly income, using the Kruskal-Wallis test and Dunn’s post-hoc test for multiple comparisons.
